# Design Strategies of 40 nm Split-Gate NOR Flash Memory Device for Low-Power Compute-in-Memory Applications

**DOI:** 10.3390/mi14091753

**Published:** 2023-09-07

**Authors:** Chan-Gi Yook, Jung Nam Kim, Yoon Kim, Wonbo Shim

**Affiliations:** 1 Department of Electrical and Information Engineering, Seoul National University of Science and Technology, Seoul 01811, Republic of Korea; yxxkchxn@naver.com; 2 School of Electrical and Computer Engineering, University of Seoul, Seoul 02504, Republic of Korea; wjdska0012@gmail.com (J.N.K.); yoonkim82@uos.ac.kr (Y.K.)

**Keywords:** compute-in-memory (CIM), NOR flash, split-gate NOR flash, device optimization, artificial intelligence, convolutional neural network, TCAD simulation

## Abstract

The existing von Neumann architecture for artificial intelligence (AI) computations suffers from excessive power consumption and memory bottlenecks. As an alternative, compute-in-memory (CIM) technology has been emerging. Among various CIM device candidates, split-gate NOR flash offers advantages such as a high density and low on-state current, enabling low-power operation, and benefiting from a high level of technological maturity. To achieve high energy efficiency and high accuracy in CIM inference chips, it is necessary to optimize device design by targeting low power consumption at the device level and surpassing baseline accuracy at the system level. In split-gate NOR flash, significant factors that can cause CIM inference accuracy drop are the device conductance variation, caused by floating gate charge variation, and a low on-off current ratio. Conductance variation generally has a trade-off relationship with the on-current, which greatly affects CIM dynamic power consumption. In this paper, we propose strategies for designing optimal devices by adjusting oxide thickness and other structural parameters. As a result of setting *T_ox,FG_* to 13.4 nm, *T_IPO_* to 4.6 nm and setting other parameters to optimal points, the design achieves erase on-current below 2 μA, program on-current below 10 pA, and off-current below 1 pA, while maintaining an inference accuracy of over 92%.

## 1. Introduction

The traditional von Neumann architecture is a structure where the processor and memory exist separately. In this architecture, data needs to be moved from memory to the processor for processing and then sent back to memory. This process leads to memory bottlenecks and large energy consumption, which are more severe in artificial intelligence (AI) computations that require large-scale data processing. Therefore, compute-in-memory (CIM) has emerged as a solution to reduce memory bottlenecks and excessive energy consumption by enabling data storage and computation within the memory simultaneously.

In CIM, various analog synaptic devices, such as static random access memory (SRAM) [1,2,3], resistive random access memory (RRAM) [4,5,6,7], and phase change memory (PCM) [8,9,10], are actively being researched as candidates to represent the weights of convolutional neural networks (CNN). However, SRAM has the disadvantage of low density due to its large cell size and power consumption caused by significant leakage power. RRAM requires a selection transistor to block the sneak path, leading to an increased unit cell size, and the high summation current during operation due to the low on-state resistance hinders low-power operation in CIM. PCM suffers from power consumption due to its high on-current value and reduced CIM inference accuracy caused by a low on-off ratio. Additionally, both ferroelectric random access memory (FeRAM) [11,12,13] and spin-transfer torque magnetic random access memory (STT-MRAM) [14,15] could be considered as candidate components for CIM architectures. However, it should be noted that FeRAM is confronted with reliability degradation attributed to variations in the capacitance of ferroelectric materials. Also, STT-MRAM devices exhibit limitations such as low sensing margins and substantial switching currents. These characteristics can compromise the accuracy and efficiency within the CIM.

Therefore, in this paper, split-gate NOR Flash is proposed as a device candidate for CIM. Split-gate NOR Flash enables the storage and processing of large-scale neural networks through its small cell size and high integration density. The low on-state current compared to other devices facilitates low-power operation in CIM. Furthermore, split-gate NOR Flash offers the benefit of utilizing mature technology. Also, compared to 3D NAND Flash [16,17,18], which is an ultra-high-density flash technology that can be used as another CIM device, the compatibility and design flexibility of the CMOS could be superior merits. Moreover, a smaller array size, low wordline and bitline capacitances, and a large on-cell current due to the non-series channel connections incur faster read speed than the NAND Flash device. Previous research has explored the split-gate NOR Flash memory-based in-memory computing technique [19]. However, the evaluation was limited to the MNIST dataset, which is a relatively simple image-classification dataset, and focused only on the system-level assessment rather than optimizing the device design.

The objective of this paper is to find the optimal device design of a split-gate NOR Flash for CIM, aiming to maintain a proper level of inference accuracy while minimizing power consumption. Key factors at the device level that can impact the inference accuracy of CIM include the device conductance variation, due to the floating-gate charge variation, and the on/off ratio. The cell design was modeled with reference to 40 nm ESF3-embedded commercial NOR flash memory technology from Silicon Storage Technology (SST Inc). [20,21]. In this study, we verify the program and erase operations at the device level and measure the on-current and off-current in the programmed and erased states using the Synopsys Sentaurus technology computer-aided design (TCAD) S-2021.06_SP1 tool [22]. Furthermore, the extracted on/off ratio and device conductance variation results obtained from TCAD are incorporated into the benchmarking framework for CIM inference called DNN+NeuroSim V1.3 [23]. This benchmarking framework utilizes the VGG-8 [24] network and supports the CIFAR-10 dataset to derive the inference accuracy as the outcome.

## 2. Design Methodology

### 2.1. Cell Structure

**Figure 1:**
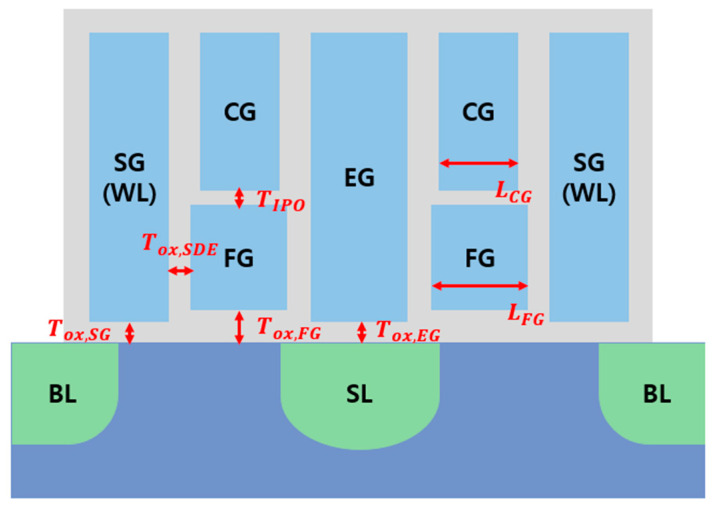
A 40 nm split-gate NOR flash cell structure and design parameters.

Figure 1 depicts the 40 nm split-gate cell structure designed in this work. The cell structure consists of a select gate (SG), a control gate (CG), a floating gate (FG), and an erase gate (EG), which are all made of polysilicon material. The gates are surrounded by oxide material, and the oxide thickness below the SG, FG, and EG is denoted as $T_{ox,SG}$, $T_{ox,FG}$, and $T_{ox,EG}$, respectively. Particularly, the oxide between the CG and the FG is referred to as interpoly oxide ($T_{IPO}$), and the oxide between the FG and the EG is referred to as side oxide ($T_{ox,SDE}$). The oxide thickness between the FG and the EG, and between the FG and the SG are equal. The gate lengths of the CG and the FG are denoted as $L_{CG}$, $L_{FG}$. The substrate is made of silicon, and an n+ doped drain is connected to the “bit line” (BL), while the n+ doped source is connected to the “source line” (SL). The SG is connected to the “word line” (WL). During the program operation, the electrons are injected to the FG by a hot carrier injection (HCI) mechanism. During the erase operation, the electrons move from the FG to the EG via a Fowler Nordheim (FN) tunneling mechanism.

### 2.2. Cell Optimization

**Figure 2:**
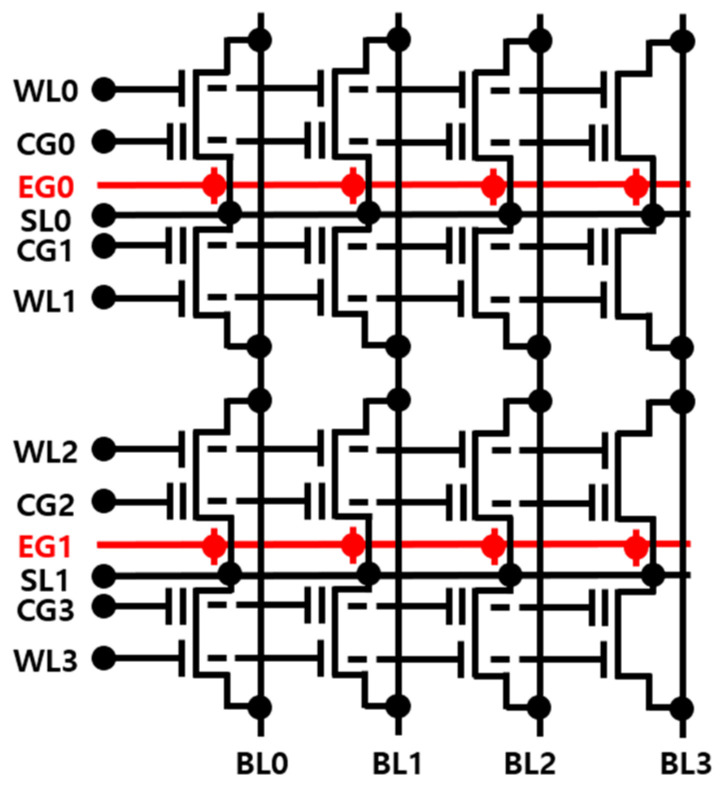
Split-gate NOR flash array schematic.

In the memory cell array, the split-gate cells store weight values by utilizing conductance states. Figure 2 illustrates a split-gate NOR flash array capable of performing analog multiply-and-accumulation (MAC) operations. The NOR flash array outputs a summation current as inputs are received during the MAC operation. During the MAC operations, every WL is activated to a high or low voltage according to the input data, causing the cells to be turned on. Therefore, to achieve low-power operation, it is necessary to minimize the on-current of the erased and programmed cells. Additionally, minimizing leakage current due to the off-current is also important.

During program and erase operations, the stored charge in the floating gate (FG) may not be uniform among cells because of various reasons, such as cell-to-cell structural variation, WL and BL RC loading, reliability, etc. Also, a non-uniform amount of injected charge in FG may occur during the programming process because of incremental step pulse programming variation, program voltage rising slope variation, etc. As a result, the charge variation could incur cell current variations. This ultimately leads to conductance variation in the cells, which is a major cause of accuracy degradation in MAC operations of CIM. It has been researched that an on-off ratio below a certain level in CIM inference operations affects accuracy drop [25].

The on-current of erased cell and conductance variation caused by charge variation generally exhibits a trade-off relationship in cell design. Therefore, an optimal cell design is required to minimize inference accuracy reduction due to charge variation while reducing power consumption. Additionally, it is necessary to validate whether the designed cell can effectively perform erase and program operations. Since the IR drop resulting from the line resistance within the array is considerably minor in comparison to the low conductance of the designed split-gate NOR Flash cell, the anticipated error stemming from this factor is expected to be negligible; hence, we disregarded it in this work. Other non-ideal effects, such as endurance and retention, are common non-ideal sources for all the nonvolatile CIM devices. However, they were not considered to emphasize accuracy degradation by charge variation in this work and remained for our later works.

### 2.3. Design Constraints

**Figure 3:**
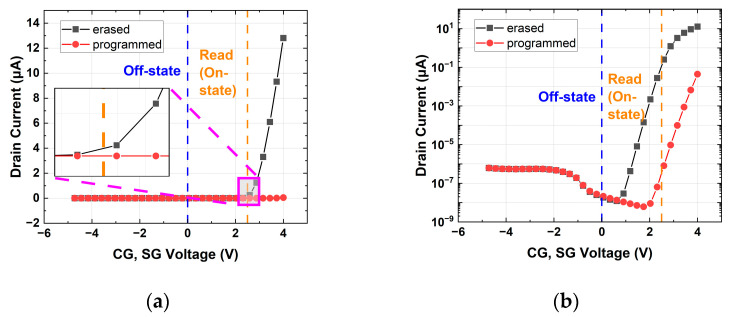
Erased and programmed split-gate NOR flash cell I–V curve: (**a**) linear scale; (**b**) log scale.

**Figure 4:**
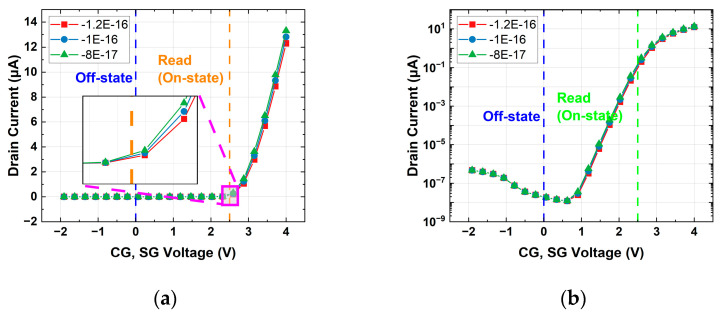
I–V curve when charge variation is applied to an erased split-gate NOR flash cell. It shows when the FG charge density is −1.2 × 10^−16^ (red), −1 × 10^−16^ (blue), and −8 × 10^−17^ (green), respectively: (**a**) linear scale; (**b**) log scale.

The bias conditions of the split-gate flash array are summarized in Table 1. To examine the current during the read operation, an I-V curve was plotted, as shown in Figure 3, by applying −5 V to 4 V simultaneously to CG and SG (WL). The program time and erase time of a cell were 10 μs and 30 μs, respectively. The charge density stored in the FG and the charge variation density when the cell was in erased or programmed states are shown in Table 2. In the erased and programmed cell, the on-state refers to the state during the read operation when $V_{dd}$ (2.5 V) was applied to the WL and CG. On the other hand, the off-state refers to the condition when no read operation was being performed, and 0 V was applied to both the WL and CG. Based on the above, the on/off ratio means the ratio of the current in on-state and current in off-state in a programmed or erased cell. Considering that the erase on-state current was much larger than the program on-state current, the charge variation in the programmed state was negligible, so we only focused on the variation of erased cells in this work. For example, if the FG charge density in the existing erased state cell was −1 × 10^−16^, the FG charge density due to charge variation ranged from −1.2 × 10^−16^ to −8 × 10^−17^. Figure 4. shows an I–V curve when a charge variation was applied to an erased split-gate NOR flash cell. By plotting the I–V curves of these cells using TCAD simulation, the variation in on-current due to the charge variation in the erased state can be obtained by comparing it with the existing cell.

The current variation in the devices leads to conductance variation, which ultimately results in the degraded accuracy of MAC operations in the CIM array. By using the DNN+NeuroSim simulation framework, the VGG-8 network can be run on the split-gate NOR flash memory-based CIM chip designed in this work to evaluate the inference accuracy on the CIFAR-10 dataset. In this paper, a pre-trained 8-bit quantized VGG-8 network using the WAGE algorithm was used [26]. WAGE quantizes both weights and activations using a fixed quantization level in the range [–1, 1], which is friendly to hardware implementation. We used a CIFAR-10 dataset consisting of 32 × 32 color images. It comprised a total of 60,000 images, with 50,000 being used for training and 10,000 for testing. For all the simulation results in our works, identical pre-trained weights were used to evaluate the inference accuracy. A 5-bit linear SAR ADC was used to sense and quantize the analog current. Although nonideal characteristics of real ADC circuits may exist, such as integral non-linearity (INL) that could affect the accuracy of the quantization, the characteristic of ADC quantization was considered as ideal in this NeuroSim simulation work. The simulation options in DNN+NeuroSim are presented in Table 3. These simulation options were designed to achieve high accuracy of over 94% without considering conductance variation, allowing for the observation of the inference accuracy degradation caused by conductance variation. The extracted on/off ratio of the cells obtained through TCAD simulation can also be introduced into DNN+NeuroSim to examine its impact on accuracy.

## 3. Experimental Results

**Figure 5:**
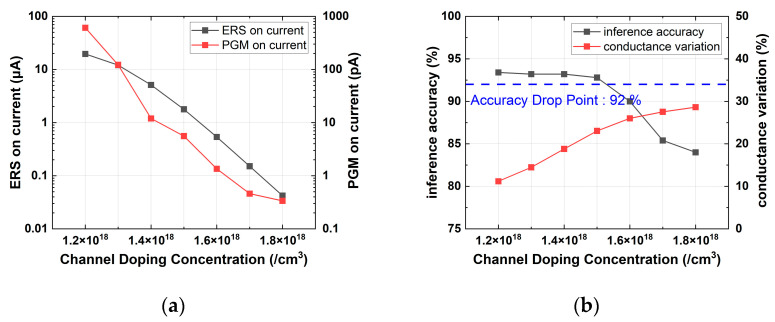
Simulation results with various channel doping concentrations: (**a**) erase and program on-current; (**b**) device conductance variation and inference accuracy.

**Figure 6:**
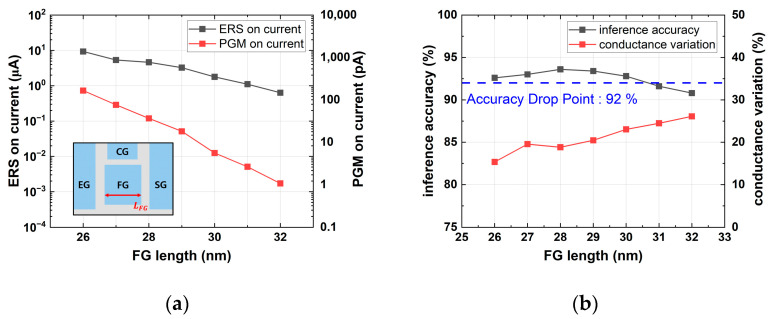
Simulation results with various $L_{FG}$ values: (**a**) erase and program on-current; (**b**) device conductance variation and inference accuracy.

**Figure 7:**
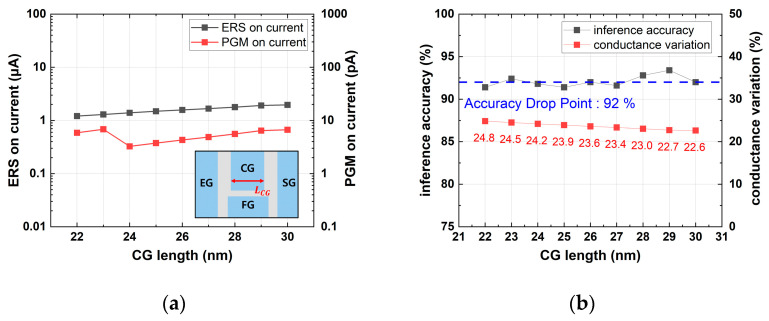
Simulation results with various $L_{CG}$ values: (**a**) erase and program on-current; (**b**) device conductance variation and inference accuracy.

**Figure 8:**
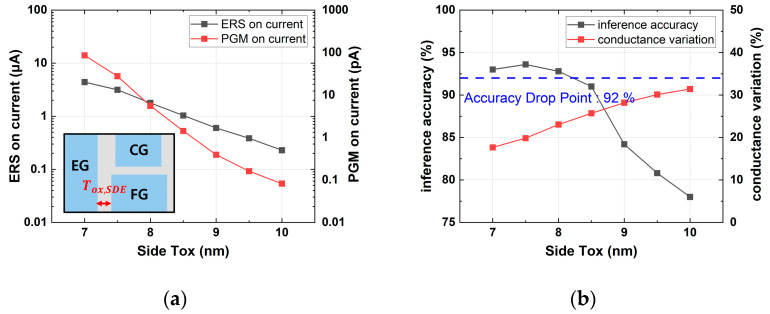
Simulation results with various $T_{ox,SDE}$ values: (**a**) erase and program on-current; (**b**) device conductance variation and inference accuracy.

**Figure 9:**
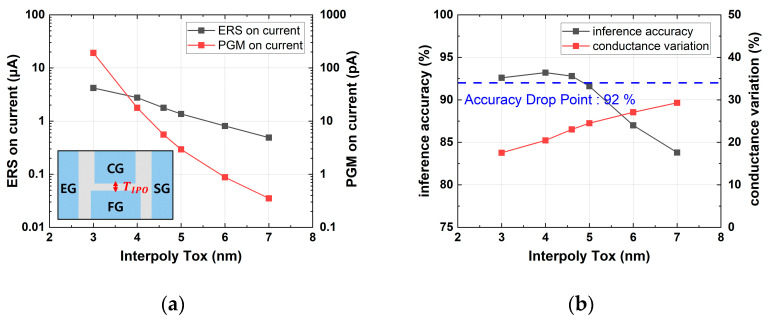
Simulation results with various $T_{IPO}$ values: (**a**) erase and program on-current; (**b**) device conductance variation and inference accuracy.

**Figure 10:**
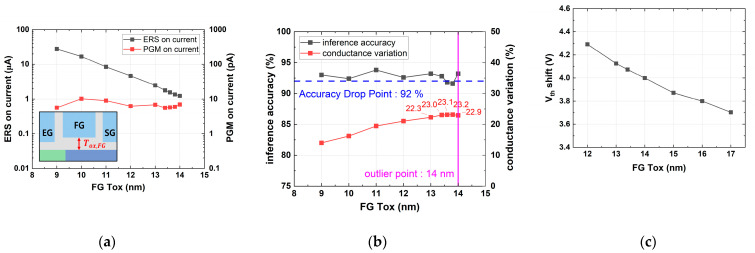
Simulation results with various $T_{ox,FG}$ values: (**a**) erase and program on-current; (**b**) device conductance variation, inference accuracy; and (**c**) threshold voltage shift from erased to programmed cell.

In this work, the split-gate flash memory cells were designed to achieve an off-current of both erased and programmed cells below 1 pA and a program on-current below 1 nA. The Figure 5, Figure 6, Figure 7, Figure 8, Figure 9 and Figure 10 depict the on-current and accuracy in the erased and programmed states, plotted by adjusting the design parameters while keeping the optimal cell as the default. When using the simulation option in Table 3, the baseline inference accuracy at an ideal variation and an on/off ratio of 100,000 was 94%. As a result of the experiment, when an accuracy drop of larger than 2% occurred due to variation, it was confirmed that the accuracy decreased rapidly even with a small variation increase. For example, from variation 0 to 23%, the accuracy tends to be around 94 to 92%, but when it increases from 23 to 25%, the accuracy drops to 89%, and at 30%, the accuracy is less than 80%. Therefore, in this paper, we defined 92% as the accuracy drop point and optimized the cell to have SW inference performance higher than the accuracy drop point. Our optimization showed that the thicker gate oxide would reduce the current variation caused by charge variation. It could interrupt the normal program or normal erase operation, so we verified it with TCAD simulation according to the operation conditions in Table 1.

Figure 5 shows that as the channel doping concentration increases, the on-current in erased and the programmed states decreased while the conductance variation increased. Since increased conductance variation leads to a decline in inference accuracy, the optimal channel doping for maintaining an accuracy of over 92% while minimizing the on-current was determined to be 1.5 × 10^18^ cm^−3^. In Figure 6, it can be observed that as $L_{FG}$ increased, the on-current in the erased and programmed states decreased and the conductance variation increased. Similar to the channel doping concentration, the optimal $L_{FG}$ for maintaining an accuracy of over 92% while minimizing the on-current was found to be 30 nm.

Figure 7 shows that as $L_{CG}$ increased, the erase on-current slightly increased, while conductance variation decreased. Since an inference accuracy over 92% can be achieved when the cell has a conductance variation less than 23.1%, the optimal $L_{CG}$ was 28 nm. In Figure 8, as $T_{ox,SDE}$ increased, the on-current during the erased and programmed states decreased, and the conductance variation increased. The optimal value for maintaining an accuracy of over 92% while minimizing the on-current was determined to be 8 nm.

Figure 9 demonstrates that as $T_{IPO}$ increased, the erase and program on-current decreased, while conductance variation increased. Consequently, the optimal point for minimizing the on-current while maintaining an accuracy of over 92% was determined to be 4.6 nm.

Figure 10 shows the simulation results with various $T_{ox,\;FG}$ values. As $T_{ox,\;FG}$ increased, the erase on-current decreased, while the program on-current remained between 5 and 11 pA. Consequently, the on/off ratio decreased gradually, while the conductance variation due to charge variation increased. As a result, there was a tendency for the accuracy to decrease below 92% when $T_{ox,\;FG}$ exceeded 13.4 nm. However, above 14 nm, the variation decreased again to below 23%. Figure 10c shows a threshold voltage ($V_{th}$) shift from erase to program according to $T_{ox,\;FG}$, when a cell was erased and then a program operation was performed. When the $T_{ox,\;FG}$ was over 14 nm, the $V_{th}$ shift tended to decrease to 4 V or less. Due to the insufficient $V_{th}$ window of erased and programmed states, we defined the outlier as $T_{ox,\;FG}\;$14 nm, and optimized cells below $T_{ox,\;FG}$ 14 nm. Therefore, the optimal $T_{ox,\;FG}$ was 13.4 nm.

## 4. Conclusions

In this paper, an analysis for the design optimization of 40 nm technology split-gate NOR Flash memory cells in CIM was presented. The optimization target was to achieve a minimal erase on-current below 2 μA while maintaining an inference accuracy of over 92% stably, considering the trade-off relationship between the on-current and conductance variation, which causes inference accuracy drop. A program on-current of less than 1 nA, an erase and program off-current of under 1 pA, and a program and erase $V_{th}$ window of more than 4 V were considered as additional conditions for optimization. The on-current is one of the main factors in CIM power consumption, so we can expect that low power consumption can be achieved by our optimum design. To design the optimal cell that meets these targets, various parameters including oxide thickness were swept and analyzed.

By completing the optimization of the design for 40 nm split-gate NOR flash cells in CIM, it was made possible to achieve low-power operation with high inference accuracy. In this paper, the single-bit per-cell NOR Flash was discussed. If it had a higher number of bits per cell, it would have a great advantage in terms of area and power consumption, but its inference accuracy would be more vulnerable to charge variation. When different networks are utilized or datasets containing larger images and more categories are employed for inference, the point at which accuracy deterioration intensifies due to charge variation may vary. Nonetheless, the design strategy presented in this paper is expected to offer a method of adjusting the trade-off between conductance variation caused by charge variation and the power consumption per cell, particularly if the baseline of the optimization is altered. Furthermore, it can be expected that the methods presented in this paper can be applied to optimize state-of-the-art split-gate NOR Flash cells with advanced technology nodes for CIM applications.

## Figures and Tables

**Table 1:** Bias condition in split-gate NOR flash array.

Electrode	Erase (V)	Program (V)	Read (V)
WL (SG)	0	1.2	$V_{dd}$
BL (drain)	0	0.3	0.8
CG	0	10	$V_{dd}$
EG	11.75	6.5	0
SL (source)	0	6.5	0

**Table 2:** FG charge density and amount of charge variation for erased and programmed state.

Charge Density (C)	
Erased state	−1 × 10^−16^
Programmed state	−1 × 10^−15^
Amount of charge variation	±2 × 10^−17^

**Table 3:** NeuroSim simulation options to evaluate the inference accuracy of the designed CIM.

NeuroSim Simulation Options	
Dataset	CIFAR-10
Network	VGG-8
Input precision	8
Weight precision	8
Activation precision	8
Memory array size	256 × 256
ADC precision	5
Bit per cell	1

## Data Availability

Data available on request due to restrictions eg privacy or ethical. The data presented in this study are available on request from the corresponding author. The data are not publicly available due to research security.
